# The clinical relevance of multiple DPYD polymorphisms on patients candidate for fluoropyrimidine based-chemotherapy. An Italian case-control study

**DOI:** 10.1038/s41416-019-0423-8

**Published:** 2019-03-12

**Authors:** Francesco Iachetta, Candida Bonelli, Alessandra Romagnani, Raffaella Zamponi, Lorenzo Tofani, Enrico Farnetti, Davide Nicoli, Angela Damato, Maria Banzi, Bruno Casali, Carmine Pinto

**Affiliations:** 1 Medical Oncology Unit, Clinical Cancer Centre, Azienda USL-IRCCS di Reggio Emilia, Reggio Emilia, Italy; 2 Molecular Biology, Oncology and Advanced Technology Unit, Azienda USL– IRCCS di Reggio Emilia, Reggio Emilia, Italy; 3 Department of Neurosciences, Psychology, Drug Research and Child Health, University of Florence, Florence, Italy

**Keywords:** Cancer genetics, Genetic predisposition to disease

## Abstract

**Background:**

Deleterious polymorphisms in the gene encoding DPD (DPYD) may result in severe reduction of DPD enzymatic activity that causes life-threatening toxicities when the standard dose of fluorouracil is used. The best panel of single-nucleotide polymorphism (SNPs) of DPYD is not well defined.

**Methods:**

In 2011, we began screening DPYD*2A in patients candidate for fluoropyrimidine-based chemotherapy. We planned a case-control study with all cases of DPYD*2A wild type who developed toxicity ≥G3 and with a cohort of patients who did not present severe toxicities. Then, we tested the additional SNPs: c.2846A>T, c.1679T>G, c.2194G>A.

**Results:**

From 2011 to 2016, we screened 1827 patients for DPD deficiency; of those, 31 subjects (1.7%) showed DPYD*2A SNP. We selected 146 subjects who developed severe toxicities (Cases) and 220 patients who experienced no or mild toxicities (Controls); 53 patients carried one of the additional SNPs: 35 subjects (66%) fell into the Cases and 18 (34%) into the Controls (*p* < 0.0001). c.2194G>A was the most frequent SNP (12.5%) and showed a correlation with neutropenia. We confirmed that c.2846A>T and c.1679T>G were related to various toxicities.

**Conclusions:**

The additional DPYD polymorphisms could enhance the prevention of fluoropyrimidine toxicity. c.2194G>A is the most frequent polymorphism and it was found to be associated with neutropenia.

## Background

Fluoropyrimidines are a class of antimetabolite drugs that are widely used in the treatment of several types of solid tumours. In the pharmacokinetics of this drug’s category, the key enzyme responsible for the catabolism of the fluoropyrimidines into inactive metabolites is the dihydropyrimidine dehydrogenase (DPD), rate-limiting step of the reaction.^1,2^

Some of the functional mutations in the dihydropyrimidine dehydrogenase gene (DPYD) are now associated with structural alterations of DPD enzyme, that induce complete inactivation or significant reduction of its activity, with consequent implication in terms of toxicity.^3–9^

One of the first single-nucleotide polymorphisms (SNPs) of the DPYD gene clearly associated with severe or even lethal toxicity is DPYD*2A (IVS14+1G>A).^10–14^ The homozygous mutation of this variant induces the complete loss of the DPD enzymatic function, the heterozygous genotype instead causes about 50% of reduction of its activity.^15,16^ However, it is a variant with low recurrence, and it has been found in about 1–2% of the population.^17^

In addition to IVS14+1G>A, other polymorphisms have been identified and to date, two DPYD genetic variants have been consistently associated with the risk of fluoropyrimidine toxicity: c.1679T>G and c.2846A>T.^17–19^

Although some guidelines suggested the pre-treatment screening of these two SNPs in association with DPYD*2A,^20^ because of their low frequency (1–2%), a debate is still on-going in the medical field about their relevance and the cost effectiveness.^21^

Furthermore, the estimated 10–15% of DPD-linked fluoropyrimidine-related adverse events (FAE)^20,22^ cannot be uniquely explained by these low-frequency variants. Therefore, additional investigations are needed to uncover other mutant genotypes that could correlate with the clinical practice.

Since 2011 in our institute, we introduced a systematic pre-treatment screening for the DPYD*2A variant for patients candidate for fluoropyrimidine therapy modifying the drug dosage in the presence of the mutant genotype. However, in some patients, we observed severe toxicities that could not be explained by the presence of this variant alone.

The objective of our case-control study was to analyse the two other variants, c.2846A>T and c.1679T>G, in subjects who have presented FAEs not DPYD*2A correlated, in order to confirm their clinical relevance and to evaluate their possible improvement of predicting toxicity when introduced in the pre-treatment screening. Furthermore, in consideration of the results of some recent studies,^5,23–25^ we evaluated the potential clinical impact of the c.2194G>A SNP, a more frequent variant of DPYD with an interesting correlation with fluoropyrimidine-related toxicity.

## Materials and methods

### Patients

In 2011 in the Oncology Units of Reggio Emilia Clinical Cancer Center, we began pre-emptive screening for DPYD*2A in all patients candidate for fluoropyrimidine-based chemotherapy. From 2011 to 2016, we screened 1827 patients, of whom 31 showed the DPYD*2A SNP variant. To investigate the clinical impact of the other DPYD SNPs, we selected patients who presented severe fluoropyrimidine-related toxicity. Adverse events (AEs) were graded according to the National Cancer Institute Common Toxicity Criteria, Version 4.0 (CTC-NCI, V4.0). We considered the following fluoropyrimidine-related AEs: diarrhoea, fatigue, nausea/vomiting, stomatitis, hand–foot syndrome, thrombocytopenia, leukopenia and neutropenia. Complete clinical information was available on 668 subjects, of whom 146 developed severe toxicity (G≥3 according to CTC-NCI, V4.0).

### Study design

We designed a case-control study (ratio 1:1.5) to retrospectively explore the clinical impact of the DPYD polymorphisms. One-hundred and forty-six cases were selected based on the occurrence of severe FAEs during treatment. The control group was formed with a cohort of individuals who did not show fluoropyrimidine intolerance or had very mild AEs (≤grade 2) according to the CTC-NCI. We identified a total of 220 subjects as controls, that matched our cases for the most important clinical features: primary tumour location, staging and patient age. For these 366 patients, we planned the analysis of three selected SNPs of DPYD: c.1679T>G and c.2846A>T, which are two established and well-documented polymorphisms correlated with fluoropyrimidine toxicity, and we also considered an additional variant, c.2194G>A, which has recently shown to have a promising role in predicting fluoropyrimidine-related toxicity.

The study was approved by an Ethics Committee. All patients provided written informed consent and the information regarding the human material was managed using anonymous numerical codes. All samples were handled in compliance with the Helsinki Declaration.

### DPYD genotyping

Genomic DNA was isolated from peripheral blood cells using the Maxwell 16 LEV Blood DNA Kit (Promega Corporation, Madison, WI, USA) according to the manufacturer’s instructions. DNA concentration and purity were determined using the Nanodrop 2000 spectrophotometer (Thermo Fisher Scientific, Waltham, MA, USA).

For polymorphism detection, we used the TaqMan SNP Genotyping Assay (Applied Biosystem, Waltham, MA, USA), which specifically recognises each single polymorphism (Assay ID: C_30633851_20; C_11985548_10; C_11372171_10; C_27530948_10).

Each genotyping assay contained two sets of primers, in order to amplify the sequence of interest, and two TaqMan probes labelled with two different dyes at the 5′ end: one probe specific for the wild-type (WT) allele and the other for the SNP variant.

PCR was performed in a CFX96 Touch Real Time Detection System (BioRad, Hercules, CA, USA), and the CFX Maestro Software was used to identify samples with different genotypes. The software displays the data as Relative Fluorescence Unit (RFU) for Allele 1/Allele 2 of each sample compared to No Template Control (NTC).

Samples that showed heterozygous or homozygous allele for the genetic variant were confirmed with PCR amplification followed by specific enzymatic digestion (RFLPs) or with direct sequencing of the amplified fragment.

### Statistical analysis

SNPs in the case-control study were analysed using logistic regression models, reporting the OR and its 95% confidence interval.

The association analysis between toxicity and SNPs (dichotomised as WT vs. homozygotes/heterozygotes) was evaluated by *χ*^2^ test (or Fisher’s exact test when necessary).

The time to toxicity (TTT) analysis, as it was used in the study of Ruzzo et al.,^23^ has been modified for our purpose. Here, we assessed the temporal occurrence of toxicity in relation to the number of chemotherapy cycles dispensed and not to time from date of randomisation in the clinical trial. TTT was defined as the number of chemotherapy cycles administered at the first appearance of toxicity. The TTT analysis was performed using the cumulative Kaplan–Meier curve and Log-Rank test.

The *p*-values were reported for two-tailed tests and the statistical significance level was set at 5%.

No sample size calculation was performed.

## Results

**Fig. 1:**
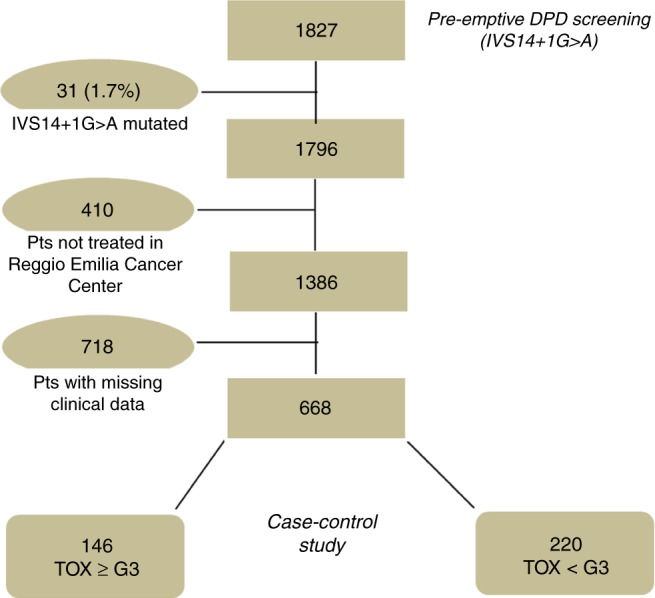
Flow chart of the study

From 2011 to 2016, we pre-emptively screened for DPD deficiency in 1827 patients. Thirty-one subjects (1.7%) were carriers of the DPYD*2A mutation and never started fluoropyrimidine-based chemotherapy. Patient medical records were examined based on their availability: for the remaining 1796 patients, 410 were not treated in our centre and 718 were excluded from the analysis due to lack of clinical data. Complete clinical information was available for 668 patients, of whom 146 (21.9%) developed severe toxicities (cases group). A control group was established with 220 patients who experienced no or mild toxicities (Fig. 1).

The patients’ clinical characteristics are reported in Table 1.

**Table 1:** Patient demographics and clinical characteristics (*N* = 366)

		Cases	%	Controls	%
Patients		146		220	
Age (years)	Median	67		65	
	Range	32–84		22–88	
Gender	M	68	46	125	57
	F	78	54	95	43
Primary tumour location	Colon	47	32	89	40
	Gastric	44	30	43	20
	Rectum	19	13	29	13
	Pancreas	10	7	15	7
	Anus	8	5	9	4
	Breast	4	3	9	4
	Oesophagus	4	3	6	3
	Bile duct	3	2	10	5
	Head and neck	3	2	7	3
	Uterine cervix	2	1	3	1
	Vulvar	2	1	0	0
Stage	I–III	88	60	132	60
	IV	58	40	88	40
Treatment setting	Neoadjuvant	11	7	23	10
	Neoadjuvant + Adjuvant	6	4	11	5
	Adjuvant	71	49	98	45
	Metastatic	58	40	88	40
Chemotherapy regimen	FU or Cape ± TT	15	10	32	15
	FU or Cape + OXA ± TT	74	50	110	50
	FU or Cape + IRI ± TT	4	3	6	3
	FU or Cape + OXA + IRI ± TT	6	4	12	5
	FU or Cape + DDP ± TT	16	11	10	4.5
	FU or Cape + DDP/OXA + TXT	11	8	19	9
	FU or Cape + mitomycin C	7	5	9	4
	FU or Cape + gemcitabine	9	6	12	5
	FU or Cape ± others	4	3	10	4.5

Others  =  anthracyclines, cyclophosphamide, nab-paclitaxel, vinorelbine.

*FU* 5-fluouracil, *Cape* capecitabine, *TT* (target therapy) cetuximab or bevacizumab or trastuzumab, *OXA* oxaliplatin, *DDP* cisplatin, *IRI* irinotecan, *TXT* taxanes (paclitaxel, docetaxel)

Cases and controls were matched in terms of primary tumour location, staging and patient age. Most patients had gastrointestinal cancer and about 50% received 5-fluouracil and oxaliplatin-based chemotherapy. Forty percent of subjects (cases and controls) were treated for metastatic disease.

Of 366 patients, 146 experienced severe toxicities (case group) and 47 presented mild toxicities. The most common AEs were neutropenia and gastrointestinal disorders.

Fifty-three patients carried a variant of one of the additional SNPs. Our study showed that DPYD polymorphisms are found more frequently in the group of cases presenting severe toxicity, with a statistically significant difference compared to controls: 35 subjects (66%) fell into the cases and 18 (34%) into the controls (OR 3.53, 95% CI 1.91–6.53. *p* < 0.0001).

c.1679T>G and c.2846A>T were present in 2 and 5 patients, respectively, of the cases group, but were absent in the controls group. A significant correlation of c.2846A>T SNP with cases presenting toxicity was confirmed (*p* = 0.0097), while c.1679T>G variant did not reach statistical relevance due to the small sample size.

c.2194G>A was the most frequent SNP, found in 46 of 366 patients (12.5%): 28 pts in the cases group (60% out of 46 pts) and 18 pts in the controls group (40%). The different distribution of this SNP in the two groups suggests a significant correlation between c.2194G>A and subjects who experienced severe toxicities (Table 2).

**Table 2:** Distribution of DPYD polymorphisms in case and control groups

		Total no.	%	Cases no.	%	Controls no.	%	*p*	OR	95% CI
DPYD SNPs	NO	313	86	111	35	202	65			
	YES	53	14	35	66	18	34	**<0.0001**	3.538	1.915–6.537
c.1679 T>G	WT	364	99.45	144	40	220	60			
	MUT	2	0.55	2	100	0	0	0.1585	7.643	0.184–317.5
c.2194G>A	WT	320	87.43	118	37	202	63			
	MUT	46	12.57	28	60	18	40	**0.0041**	2.618	1.364–5.026
c.2846A>T	WT	361	98.63	141	39	220	61			
	MUT	5	1.37	5	100	0	0	**0.0097**	17.157	0.714–412.4

Bold values are statistical significance values

In the association analysis, we evaluated the possible correlation of polymorphisms with AEs grouped as: haematological, neutropenia, intestinal and others (skin, cardiac, hepatic, fatigue, renal, mucositis, thromboembolism, ischaemia, pancreatitis). The single toxicities are reported in Supplementary Table S1.

We confirmed that c.2846A>T (1.37%, 5 out of 366 pts) was related to neutropenia (*p* = 0.0141) and other toxicity (*p* = 0.0049), while c.1679T>G (0.55%, 2 out of 366 pts) showed only gastrointestinal toxicity (*p* = 0.0027).

A statistically significant association was found between c.2194G>A and neutropenia. In particular, as shown in Table 3, neutropenia was observed in 50% of the patients carrying c.2194G>A (23 out of 46) vs. 21% of the patients carrying WT genotype (67 out of 320) (OR = 3.75, 95% CI 1.98–7.10; *p* < 0.0001).

**Table 3:** c.2194 polymorphism and its correlation with toxicity

		Total n.	%	c.2194G/G no.	%	c.2194 (G/A)+(A/A) no.	%	*p*	OR	95% CI
All toxicities	NO	192	52	180	56	12	26			
	YES	174	48	140	44	34	74	**0.0001**	3.548	1.779–7.076
Haematological	NO	275	75	253	79	22	48			
	YES	91	25	67	21	24	52	**<0.0001**	4.090	2.161–7.741
Neutropenia	NO	276	75	253	79	23	50			
	YES	90	25	67	21	23	50	**<0.0001**	3.756	1.986–7.106
Intestinal	NO	299	82	260	81	39	85			
	YES	67	18	60	19	7	15	0.5624	0.817	0.354–1.889
Others	NO	335	92	293	92	42	91			
	YES	31	8	27	8	4	9	0.9531	1.130	0.393–3.253

Others = skin, cardiac, hepatic, fatigue, renal, mucositis, thromboembolism, ischaemia, pancreatitis

In the TTT analysis, we found a significant difference of occurrence of toxicity in patients with one of the three SNPs vs. WT patients (median TTT 5 vs. 11 cycles; *p* < 0.0001). In particular, the very early occurrence of AEs was reported in patients carrying the c.1679T>G variant, with a median TTT of 2 vs. 8 cycles (*p* = 0.02) compared to the WT, and in patients with the c.2846A>T SNP, with a median TTT of 1 vs. 8 cycles (*p* < 0.0001).

**Fig. 2:**
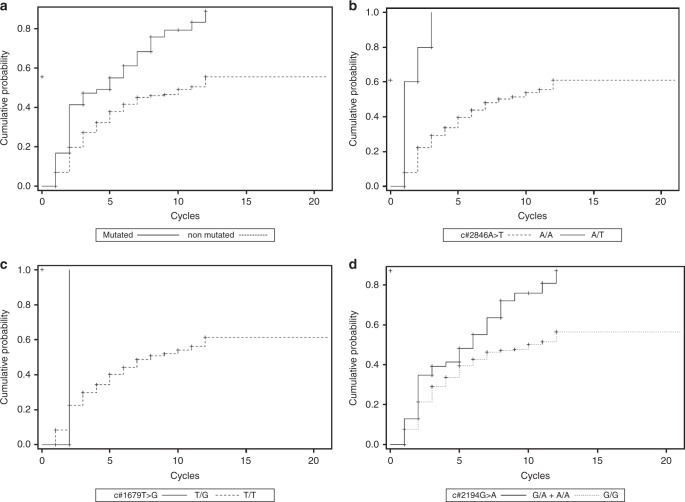
Time to toxicity (TTT) analysis (Kaplan–Meier curves). **a** TTT curves in mutated vs. non-mutated patients. **b** TTT curves of c.2846A>T. **c** TTT curves of c.1679T>G. **d** TTT curves of c.2194G>A

Moreover, patients carrying the c.2194G>A variant experienced an even earlier onset of AE compared to the WT as shown from a median TTT of 6 cycles vs. 10 (*p* = 0.0022), with a significant difference of Kaplan–Meier curves between the two groups (Fig. 2).

## Discussion

Substantial evidence demonstrated the clinical impact of DPYD*2A in the screening for fluoropyrimidine-related toxicity, and the current guidelines^20^ recommend a reduced dose of therapeutic drug in patients who are carriers of this variant. In our centre, since 2011, we have begun a pre-therapeutic screening for DPYD*2A in all patients candidate for fluoropyrimidine-based chemotherapy, and in clinical practice, we implemented a dose reduction or alternative treatment in mutated patients. However, the results of our analysis showed that this rare deleterious variant alone is indeed not able to explain the overall estimated contribution of functional DPYD variants in causing severe fluoropyrimidine toxicity. Exploiting the improvement in the sensitivity of DPYD genotyping, we focused our studies on two other well-known detrimental variants (c.2846A>T and c.1679T>G) and one with an emerging predictive role (c.2194G>A).

With large monocentric clinical records available, we designed a case-control study excluding patients who are carriers of DPYD*2A. Our study showed that patients who are carriers of one of the three DPYD variants, c.2846A>T, c.1679T>G or c.2194G>A, had a significantly increased risk of fluoropyrimidine-associated toxicity, confirming the clinical relevance of pre-treatment screening for these DPYD polymorphisms. In fact, in our study 66% of the patients carrying one of these DPYD variants presented severe toxicity and we showed that DPYD polymorphisms are found more frequently in the group of cases, with a statistically significant difference compared to controls.

AE correlation and clinical validity for both c.2846A>T and c.1679T>G SNPs are very well established in the literature.^17–19^

The clinical relevance of c.2846A>T variant was documented in three meta-analyses where they all showed convincing data of an association between this variant and the development of fluoropyrimidine-related toxicities. The first one by Terrazzino et al.,^18^ compared 15 studies with 4094 patients for DPYD*2A and 2308 patients for c.2846A>T. The second meta-analysis, performed by Rosmarin et al.,^19^ included data on 4855 patients from 17 studies where they described eight different DPYD variants, including DPYD*2A and c.2846A>T. The third meta-analysis, by Meulendijks et al.,^17^ included data on 7365 patients from eight studies and confirmed the association between severe toxicity and the variants DPYD*2A and c.2846A>T.

Our results reaffirm the clinical relevance of c.2846A>T, showing a significant correlation of this polymorphism with the cases group that presented severe toxicity. Furthermore, we found a statistical association of this variant with specific fluoropyrimidine-related AES, like neutropenia and other toxicities.

For c.1679T>G, the meta-analysis of Meulendijks et al.^17^ considered five studies with a total of 5616 patients, of whom only 11 subjects with this mutation were described.

The data showed that patients carrying this specific variant have an increased risk of global severe AE of about four times and risk of haematological and gastrointestinal toxicities increased by 9.8 and 5.7 times, respectively.^17^ Based on the available functional data, where a heterozygous genotype is expected to result in a 40–50% decrease in DPD activity, similar to the effect of DPYD*2A, and the clinical data presented in this study, the authors recommended a dose reduction of 50% in patients with c.1679T>G genotype, in line with the recommendation by the Clinical Pharmacogenetics Implementation Consortium.^20^

In our case-control study, we found two patients carrying the c.1679 T>G variant, and who were present in the cases group but absent in the controls group. Although there was an evident correlation between this polymorphism and FAEs, we could not confirm statistical relevance due to the small sample size. However, in line with the results reported in the meta-analyses, we found a significant association between c.1679T>G genotype and gastrointestinal toxicity, but not with other toxicities.

The clinical impact of c.2194G>A SNP is still controversial, although recent studies have shown promising relevance in the correlation with fluoropyrimidine-related toxicity.^5,23–25^

In the pharmacogenetics analysis on 927 patients of the QUASAR II trial,^19^ c.2194G>A variant was not found to be associated with fluoropyrimidine toxicity although it should be considered that this study was performed in patients treated with capecitabine mono-chemotherapy only.

Likewise, in the prospective study by Schwab et al.,^12^ which included 683 patients with different tumour types treated with fluorouracil monotherapy regimes, no association was found between this variant and severe FAEs.

In contrast, a recent case-cohort analysis carried out in 568 previously untreated patients with advanced colon cancer participating in the CAIRO trial and assigned to capecitabine therapy combined with oxaliplatin, and bevacizumab with or without cetuximab, the c.2194G>A variant was significantly associated with grade 3–4 diarrhoea but with a rather low predictive value of 41%.^25^

In the PETACC-8 trials, the c.2194G>A SNP was associated with increased fluorouracil-related AEs, including haematologic AEs and neutropenia (OR 1.8, 95% CI 1.3–2.4) in 1545 colorectal cancer (CRC) patients who received standard adjuvant FOLFOX4 or FOLFOX4 in combination with cetuximab.^24^

The pharmacogenetics study of the TOSCA trial,^23^ in which colon cancer patients were enroled for 3 or 6 months of either FOLFOX or XELOX adjuvant chemotherapy, tested ten DPYP variants in correlation with >grade 3 FAEs. In the association analysis of 508 patients, FAEs occurred more frequently in c.2194G>A carriers, and this DPYD variant showed detrimental effects on the time to neutropenia. The TTT analysis showed the clinical impact of the c.2194G>A SNP with a significantly shorter TTT occurrence in DPYD variant carriers. Median TTTs for c.2194G>A GG, GA and AA genotype carriers were 7.0, 3.0 and 2.1 months, respectively.

Our data confirmed the association between the c.2194G>A SNP and severe toxicity (Table 3), and resulted in line with both the PETACC8 and TOSCA studies in terms of the significant correlation with haematologic toxicity.^23,24^ In particular, we found a statistically significant association between this SNP and severe neutropenia, which was observed in 50% of the patients carrying c.2194G>A (23 out of 46) vs. 21% of the patients with WT genotype (67 out of 320) (*p* < 0.0001).

Moreover, we performed a TTT analysis that considered the temporal aspect of the emerging toxicity in relation to the number of cycles of therapy. This analysis avoids considering only the early toxicity occurring in the first cycles of therapy. In fact, some DPYD variants may not induce a dramatic loss of enzymatic function, and therefore the TTT approach may be more sensitive for detecting the risk of toxicity determined by DPYD variants with moderate functional effects.

The significant difference in Kaplan–Meier curves between DPYD variant carriers and WT subjects graphically showed the potential clinical impact of pre-emptive screening for these variants. The time of toxicity occurrence was significantly shorter in patients who are carriers of one of the three SNPs considered. In detail, patients carrying c.1679T>G and c.2846A>T SNPs showed very early occurrence of AEs. As shown in Fig. 2, the toxicity appeared in the first 2–3 cycles of therapy in patients carrying these deleterious polymorphisms, highlighting their dramatic clinical impact as already reported in the literature.^7–9,17–20,22^

Patients with the c.2194G>A variant showed an earlier onset of toxicity compared to WT subjects (median TTT of 6 vs. 10 cycles, *p* = 0.0022), with a significant difference in Kaplan–Meier curves between the two groups. However, the temporal occurrence of AEs is different compared to other SNPs. In fact, the clinical impact of this variant is more evident in a late phase of therapy. This event could be explained by a moderate reduction of enzymatic function correlated with this SNP that arises in the advanced cycles of therapy by cumulative effect. As reported in the study by Offer et al.,^16^ the estimated reduction of DPD enzyme activity in the presence of c.2194G>A polymorphism is about 15–20%.

Moreover, the moderate reduction in the enzymatic function determined by c.2194G>A variant can explain why the detrimental effect of this SNP is not evident in patients treated with fluoropyrimidine alone,^12,19^ but it emerges during a poly-chemotherapy treatment.^23–25^

Our data are indeed in line with previous studies and support the hypothesis that the synergistic effects of the poly-chemotherapy, together with fluorouracil, make this DPD enzymatic deficit more evident and potentially dangerous.

In conclusion, we confirmed the clinical relevance of the two deleterious variants of DPYD, the c.2846A>T and the c.1679T>G, in accordance with the literature, and we propose to introduce into clinical practice the evaluation of these genotypic variants in pre-treatment screening, in addition to the DPYD*2A tests. Besides, our data suggest a predictive role for the c.2194G>A genotype in the fluoropyrimidine-related toxicity.

## Supplementary information

Supplementary Information

## **Data availability:**

All data are available at Reggio Emilia Cancer Center for consultation.

## References

[1] Longley DB, Harkin DP, Johnston PG (2003). 5-fluorouracil: mechanisms of action and clinical strategies. Nat. Rev. Cancer.

[2] Diasio RB, Harris BE (1989). Clinical pharmacology of 5-fluorouracil. Clin. Pharmacokinet..

[3] Van Kuilenburg AB, van Lenthe H, Blom MJ, Mul EP, Van Gennip AH (1999). Profound variation in dihydropyrimidine dehydrogenase activity in human blood cells: major implications for the detection of partly deficient patients. Br. J. Cancer.

[4] Raida M (2001). Prevalence of a common point mutation in the dihydropyrimidine dehydrogenase (DPD) gene within the 5’-splice donor site of intron 14 in patients with severe 5-fluorouracil (5-FU)- related toxicity compared with controls. Clin. Cancer Res..

[5] Del ReM (2015). Discovery of novel mutations in the dihydropyrimidine dehydrogenase gene associated with toxicity of fluoropyrimidines and viewpoint on preemptive pharmacogenetic screening in patients. EPMA J..

[6] Lee AM (2014). DPYD variants as predictors of 5-fluorouracil toxicity in adjuvant colon cancer treatment (NCCTG N0147). J. Natl. Cancer Inst..

[7] Gentile G (2015). Genotype-phenotype correlations in 5-fluorouracil metabolism: a candidate DPYD haplotype to improve toxicity prediction. Pharmacogenomics J..

[8] Henricks LM (2015). Translating DPYD genotype into DPD phenotype: using the DPYD gene activity score. Pharmacogenomics.

[9] Toffoli G (2015). Clinical validity of a DPYD-based pharmacogenetic test to predict severe toxicity to fluoropyrimidines. Int. J. Cancer.

[10] van Kuilenburg AB (2001). Lethal outcome of a patient with a complete dihydropyrimidine dehydrogenase (DPD) deficiency after administration of 5-fluorouracil: frequency of the common IVS14+1G>A mutation causing DPD deficiency. Clin. Cancer Res..

[11] van Kuilenburg AB, Meinsma R, Zoetekouw L, Van Gennip AH (2002). Increased risk of grade IV neutropenia after administration of 5-fluorouracil due to a dihydropyrimidine dehydrogenase deficiency: high prevalence of the IVS14+1g>a mutation. Int. J. Cancer.

[12] Schwab M (2008). Role of genetic and nongenetic factors for fluorouracil treatment related severe toxicity: a prospective clinical trial by the German 5-FU Toxicity Study Group. J. Clin. Oncol..

[13] Saif MW, Ezzeldin H, Vance K, Sellers S, Diasio RB (2007). DPYD*2A mutation: the most common mutation associated with DPD deficiency. Cancer Chemother. Pharmacol..

[14] Braun MS (2009). Association of molecular markers with toxicity outcomes in a randomized trial of chemotherapy for advanced colorectal cancer: the FOCUS trial. J. Clin. Oncol..

[15] Offer SM, Wegner NJ, Fossum C, Wang K, Diasio RB (2013). Phenotypic profiling of DPYD variations relevant to 5-fluorouracil sensitivity using real-time cellular analysis and in vitro measurement of enzyme activity. Cancer Res..

[16] Offer SM (2014). Comparative functional analysis of DPYD variants of potential clinical relevance to dihydropyrimidine dehydrogenase activity. Cancer Res..

[17] Meulendjiks D (2015). Clinical relevance of DPYD variants c.1679T>G, c.1236G>A/HapB3, and c.1601G>A as predictors of severe fluoropyrimidine-associated toxicity: a systematic review and meta-analysis of individual patient data. Lancet Oncol..

[18] Terrazzino S (2013). DPYD IVS14+1G>A and 2846A>T genotyping for the prediction of severe fluoropyrimidine-related toxicity: a meta-analysis. Pharmacogenomics.

[19] Rosmarin D (2014). Genetic markers of toxicity from capecitabine and other fluorouracil-based regimens: investigation in the QUASAR2 study, systematic review, and meta-analysis. J. Clin. Oncol..

[20] Caudle KE (2013). Clinical Pharmacogenetics Implementation Consortium guidelines for dihydropyrimidine dehydrogenase genotype and fluoropyrimidine dosing. Clin. Pharmacol. Ther..

[21] Deenen MJ (2016). Upfront genotyping of DPYD*2A to individualize fluoropyrimidine therapy: a safety and cost analysis. J. Clin. Oncol..

[22] Meulendijks D, Cats A, Beijnen JH, Schellens JH (2016). Improving safety of fluoropyrimidine chemotherapy by individualizing treatment based on dihydropyrimidine dehydrogenase activity-ready for clinical practice?. Cancer Treat. Rev..

[23] Ruzzo A (2017). Dihydropyrimidine dehydrogenase pharmacogenetics for predicting fluoropyrimidine-related toxicity in the randomised, phase III adjuvant TOSCA trial in high-risk colon cancer patients. Br. J. Cancer.

[24] Boige Valérie, Vincent Marc, Alexandre Philippe, Tejpar Sabine, Landolfi Stefania, Le Malicot Karine, Greil Richard, Cuyle Pieter Jan, Yilmaz Mette, Faroux Roger, Matzdorff Axel, Salazar Ramon, Lepage Côme, Taieb Julien, Laurent-Puig Pierre (2016). DPYDGenotyping to Predict Adverse Events Following Treatment With Fluorouracil-Based Adjuvant Chemotherapy in Patients With Stage III Colon Cancer. JAMA Oncology.

[25] Deenen MJ (2011). Relationship between single nucleotide polymorphisms and haplotypes in DPYD and toxicity and efficacy of capecitabine in advanced colorectal cancer. Clin. Cancer Res..

