# Supplementary material for: The clinical relevance of multiple DPYD polymorphisms on patients candidate for fluoropyrimidine based-chemotherapy. An Italian case-control study
Source: Br J Cancer. 2019 Mar 12;120(8):834–9. doi: 10.1038/s41416-019-0423-8 (PMC6474277; doi:10.1038/s41416-019-0423-8)
Supplement: Supplementary file 1 — Supplementary Information [file 41416_2019_423_MOESM1_ESM.doc]

**Table S1. Correlation between DPYD polymorphisms and “other” toxicities.**

|  |  | **Total**  **No.** | **%** | **WT** | **%** | **DPYD SNPs** | **%** | **p** | **OR** | **95% CI** |
| --- | --- | --- | --- | --- | --- | --- | --- | --- | --- | --- |
| **Other Toxicities** | NO | 335 | 91.5 | 287 | 91.7 | 48 | 90.5 |  |  |  |
|  | YES | 31 | 8.5 | 26 | 8.3 | 5 | 9.5 | 0.183 | 1.83 | 0.747-4.496 |
| **Fatigue** | NO | 362 | 98.9 | 310 | 99 | 52 | 98.1 |  |  |  |
|  | YES | 4 | 1.1 | 3 | 1 | 1 | 1.9 | NS |  |  |
| **Mucositis** | NO | 359 | 98 | 306 | 97.7 | 53 | 100 |  |  |  |
|  | YES | 7 | 2 | 7 | 2.3 | 0 | 0 | NS |  |  |
| **Hand foot syndrome** | NO | 360 | 98.3 | 308 | 98.4 | 52 | 98.1 |  |  |  |
|  | YES | 6 | 1.7 | 5 | 1.6 | 1 | 1.9 | NS |  |  |
| **Hepatic** | NO | 358 | 97.8 | 307 | 98 | 51 | 96.2 |  |  |  |
|  | YES | 8 | 2.2 | 6 | 2 | 2 | 3.8 | NS |  |  |
| **Renal** | NO | 365 | 99.7 | 313 | 100 | 52 | 98.1 |  |  |  |
|  | YES | 1 | 0.3 | 0 | 0 | 1 | 1.9 | NS |  |  |
| **Tromboembolism** | NO | 365 | 99.7 | 312 | 99.7 | 53 | 100 |  |  |  |
|  | YES | 1 | 0.3 | 1 | 0.3 | 0 | 0 | NS |  |  |
| **Ischemia** | NO | 363 | 99.1 | 310 | 99 | 53 | 100 |  |  |  |
|  | YES | 3 | 0.9 | 3 | 1 | 0 | 0 | NS |  |  |
| **Pancreatitis** | NO | 365 | 99.7 | 312 | 99.7 | 53 | 100 |  |  |  |
|  | YES | 1 | 0.3 | 1 | 0.3 | 0 | 0 | NS |  |  |

NS not significant
